# Zika antiviral chemotherapy: identification of drugs and promising starting points for drug discovery from an FDA-approved library

**DOI:** 10.12688/f1000research.9648.1

**Published:** 2016-10-14

**Authors:** Bruno S. Pascoalino, Gilles Courtemanche, Marli T. Cordeiro, Laura H. V. G. Gil, Lucio Freitas-Junior

**Affiliations:** 1 Laboratório Nacional de Biociências, Centro Nacional de Pesquisa em Energia e Materiais, Campinas-SP, 10000, Brazil; 2 BIOASTER, Paris, 75015, France; 3 Centro de Pesquisas Aggeu Magalhães, Fundação Oswaldo Cruz -Fiocruz, Recife/PE, Brazil; 4 Present Address: Instituto Butantan, São Paulo-SP, 1500, Brazil

**Keywords:** Zika, High content screening drug discovery, FDA-approved drugs

## Abstract

**Background**

The recent epidemics of Zika virus (ZIKV) implicated it as the cause of serious and potentially lethal congenital conditions such microcephaly and other central nervous system defects, as well as the development of the Guillain-Barré syndrome in otherwise healthy patients. Recent findings showed that anti-Dengue antibodies are capable of amplifying ZIKV infection by a mechanism similar to antibody-dependent enhancement, increasing the severity of the disease. This scenario becomes potentially catastrophic when the global burden of Dengue and the advent of the newly approved anti-Dengue vaccines in the near future are taken into account. Thus, antiviral chemotherapy should be pursued as a priority strategy to control the spread of the virus and prevent the complications associated with Zika.

**Methods**

Here we describe a fast and reliable cell-based, high-content screening assay for discovery of anti-ZIKV compounds. This methodology has been used to screen the National Institute of Health Clinical Collection compound library, a small collection of FDA-approved drugs.

**Results and conclusion**

From 725 FDA-approved compounds triaged, 29 (4%) were found to have anti-Zika virus activity, of which 22 had confirmed (76% of confirmation) by dose-response curves. Five candidates presented selective activity against ZIKV infection and replication in a human cell line. These hits have abroad spectrum of chemotypes and therapeutic uses, offering valuable opportunities for selection of leads for antiviral drug discovery.

## Introduction

Zika virus (ZIKV) is a mosquito-borne virus transmitted by *Aedes* sp. mosquitoes across tropical and subtropical regions around the world. It is a positive single strand RNA flavivirus responsible for, in most of cases, asymptomatic infections. The most common symptoms of Zika are very similar to Dengue fever including headache, muscle and joint pain, mild fever, rash, and inflammation of the underside of the eyelid ^1^ and, given the commonality of such symptoms, the diagnose is usually imprecise. The virus was first reported in Uganda in 1947 and 60 years after its discovery, only 15 cases were documented until the start of the current epidemics in Americas, mainly in Brazil ^2^. Although ZIKV was first isolated nearly 70 years ago, very little is known about the virus biology, as most of the cases likely remained unreported and the transmission had been sporadic and silent for most of time ^3^.

The Latin America Zika epidemic drew attention especially due to the related cases of microcephaly. Since 2014, the number of microcephaly cases in Brazil increased 20 times and its incidence overlapped with Zika epidemic areas ^2^. Moreover, recent work has demonstrated that Zika infection impairs the growth of neurospheres ^4^ and causes birth defects in mice ^5^, indicating the virus influence in the fetal development. Although the more recent Zika outbreaks suggests that *Aedes aegypti* is the main vector, it has been shown that other mosquito species are capable of carrying and thus possibly transmitting the virus ^6^ – for example, ZIKV was isolated from the ubiquitous *Culex* species, which is also present in countries of milder climates. But the importance of this mosquito as a potential disease vector is still not understood ^3^. Furthermore, sexual transmission of Zika virus was already reported in temperate countries without mosquito vectors ^6^ suggesting that Zika transmission could eventually be established outside tropical areas.

Recent studies call attention for the risk of pre-immunity to Dengue leading to complications during Zika. Anti-Dengue antibodies could enhance the infection in Zika ^7^, most likely by a mechanism known as antibody-dependent enhancement (ADE), which is also the pathophysiological mechanism that causes severe Dengue. The Dengue epidemics persists in the tropical and sub-tropical areas around the globe, and combined with the upcoming introduction of newly developed anti-Dengue vaccines ^8, 9^, it could lead to a potentially catastrophic scenario when Zika complications due to ADE are considered. For this reason, vaccines should not be the only control strategy to pursue against epidemic flaviviruses. Efforts must be focused in the development of novel approaches to control the pathogens, instead of just depending on the vector control and palliative care to ease the disease symptoms.

High content screening (HCS) was recently used for discovery of inhibitors of Dengue virus (DENV) and Chikungunya virus (CHIKV) infection ^10, 11^. This is a cell-based and innovative image-based assay using libraries of small molecules against the viruses to identify compounds that possess antiviral activity during infection of a human host cell. The advantage of HCS over others High-throughput screening (HTS) assays (such as target-based) is that the amount of information that can be generated from images of a single treatment is not limited to a single value. Aside from the degree of viral infection and cell viability, other relevant information can be extracted from images such as morphological changes in host cell, protein localization, among others ^12^. Another advantage is that HCS precludes the need for a validated target, as compounds can be screened against all putative molecular targets at a single experiment, in a physiologically relevant condition. This condition becomes a considerable advantage in the case of Zika, in which both viral and host targets remain to be discovered. Thus, cell-based screening is a viable strategy to rapidly advance drug discovery for Zika.

Drug repurposing is a well-known strategy by the pharmaceutical industry, that speeds-up the drug discovery process. Also known as drug repositioning, it is basically the use of known drugs or compounds to treat new indications. The obvious advantage of drug repurposing over the traditional drug development is the gain in time and the lower costs, since the repurposed drug has already been approved for clinical use. For this reason, in addition to quickly enabling the start of clinical trials for a different therapeutic use, the risk of failure due to adverse toxicology is greatly reduced. Besides drug repurposing, these compounds can also serve as starting material for the development of leads for new therapeutic purposes.

Here we describe a high content screening methodology for the discovery of inhibitors of ZIKV infection applied in a drug repurposing context. This assay was used to screen a library of FDA-approved drugs, resulting in the identification of five compounds with selective activity against ZIKV in human cells.

## Methods

### Zika virus (ZIKV)

The Zika virus (KX197192.1) used in this project was isolated from a patient in Pernambuco-Brazil in 2015.

### Huh7

The human hepatome cell Huh7 (JCRB0403), obtained from the Japanese Cell Bank, was cultivated in DMEM F-12 media (Sigma-Aldrich) supplemented with 10% fetal bovine serum (FBS) (Sigma-Aldrich), 100 units/mL of Penicillin and 100 μg/mL of Streptomycin (Sigma-Aldrich), at 37°C, 5% CO _2_.

### C636

The *Aedes albopictus* cell C636, kindly provided by Dr. Amílcar Tanuri from Universidade Federal do Rio de Janeiro, was cultivated in Leibovitz L-15 media (Sigma-Aldrich) supplemented with 10% FBS (Sigma-Aldrich), 0.26% tryptose phosphate (Sigma-Aldrich), 100 units/mL of Penicillin and 100 μg/mL of Streptomycin (Sigma-Aldrich), at 28°C ^13^.

### Hybridoma cells D1-4G2-4-15 (HB-112)

The mouse hybridoma cells D1-4G2-4-15 (HB-112), obtained from Rio de Janeiro Cell Bank, was cultivated in DMEM F-12 media (Sigma-Aldrich), supplemented with 10% FBS (Sigma-Aldrich), 100 units/mL of Penicillin and 100 μg/mL of Streptomycin (Sigma-Aldrich), at 37°C, 5% CO _2_. Exponentially growing hybridoma cells were used to produce ascitic fluid as described by Yokoyama *et al.* ^14^.

### Viral propagation and quantification

Zika viruses were used to infect C636 cells at 80% confluency at a multiplicity of infection (MOI) of 0.01 for 96 h. The supernatant was harvested, aliquoted in sterile conical tubes and frozen at -80°C. The obtained viruses were quantified by plaque assay using Huh7 cells, as described for Dengue virus by Medina *et al*. ^15^.

### Compound libraries and reference compound

The NIH Clinical Collection compound library (Evotec) was used. The human recombinant Interferon α 2A (Sigma-Aldrich) was used as reference compound. The compounds were diluted in 100% dimethylsulfoxide (DMSO) (Sigma-Aldrich), with the exception of IFNα2A (Thermo Scientific) that was prepared in Dulbecco's Phosphate-buffered saline (DPBS) (Sigma-Aldrich) containing 0.5% (W/V) bovine albumin (Sigma-Aldrich).

### ZIKV compound screening assay

The NIH Clinical Collection compound library (Evotec) was screened against ZIKV at 20 μM in 1% DMSO. MOCK-infected Huh7 and IFNα2A (1.55 nM) were used as positive controls, and the 1% DMSO (vehicle)-treated cells were used as negative control. In each run, a 10-point dose-response curve of the reference compound IFNα2A, starting at 1.55 nM and diluted in a factor of 2, was also used for assay quality control. The compounds were diluted 16.6× in DPBS 1× in the µClear Black 384-well plates (Greiner Bio-One) for a final volume of 10 µL of compound at 6% DMSO. After that, 50 μL of a mixture of Huh7 cells at 6 × 10 ^4^ cells/mL and ZIKV at a MOI of 0.5, were added in each well of the plate resulting in a final concentration of 1% DMSO and a final volume of 60 μl/well. After 72 h of incubation at 37°C and 5% CO _2_, the cells were submitted to indirect immunofluorescence (IF) protocol as described below. The primary screening was performed in two independent experiments and the confirmation ratio was calculated by the number of common hits in both assays divided by the total number of hits of the first assay using Pearson test in Graphpad Prism software, version 6. Scatter-plot distribution of the entire screening was generated using Spotfire 7.0 (TIBCO).

### Detection of infected cells by indirect immunofluorescence

The Huh7 cells were fixed with 4% (w/v) (PFA) (Sigma-Aldrich) for 30 min at room temperature, treated with 0.25% (v/v) Triton-X for 15 min and incubated with the primary monoclonal antibody D1-4G2-4-15 (HB-112) prepared in DPBS containing 2.5% FBS at 37°C for 2 h. After two wash steps with DPBS, plates were incubated with AlexaFluor594 conjugated goat anti-mouse IgG (Thermo Scientific) and 5 μg/mL of DAPI (4, 6 diamidino-2-phenylindole) (Sigma-Aldrich) in DPBS at 37°C temperature for 1 h, and then washed again twice with DPBS. After the final washing, digital images were acquired using a high content imaging system, the Operetta (Perkin Elmer). The digital images were taken from four different fields of each well at 20× magnification.

### Data normalization and assay quality control

**Figure 1.**
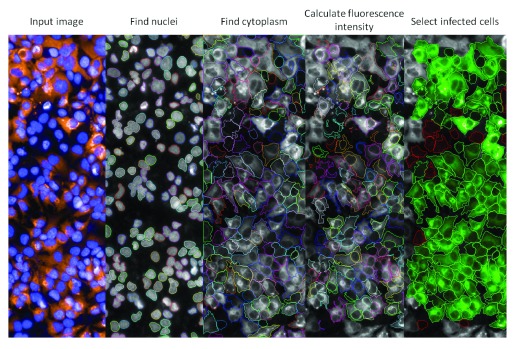
Interface of image processing and analysis developed for the Zika High content screening. The acquired images (input image) have the individual cells selected (find nuclei and find cytoplasm), the intensity of virus labeling is calculated (calculate fluorescence intensity) and the cells with signal higher than the defined threshold are selected as virus infected (select infected cells). Blue: Cell nuclei labeled with Dapi; Orange: D1-4G2-4-15 monoclonal antibody labeling.

The acquired images were analyzed with the High Content Analysis (HCA) software Columbus (Perkin Elmer) for identification, segmentation and quantification of host cell nucleus, cytoplasm and intracellular virus labeling with the specific antibody ( Figure 1). The HCA provides as output data for all images from one well the total number of cells and total number of infected cells. For the purpose of this study, the infection ratio (IR) was defined as the ratio between the total number of infected cells in all images from the well and the total number of cells in all images from the same well. The raw data for IR values were normalized to negative (infected cells, DMSO-treated) and positive controls (infected cells treated with Interferon α 2A at a concentration of 1.55 nM) to determine the normalized antiviral activity, according to the equation below:

(i) Normalized Activity (NA) = [1- (Av. IRN – Av. IRT)/(Av. IRN – Av. IRP)] × 100

where:

Av. IRN: average infection ratio of negative control wells

Av. IRP: average infection ratio of positive control wells

Av. IRT: average infection ratio of test compound wells (in a given concentration)

**Figure 2.**
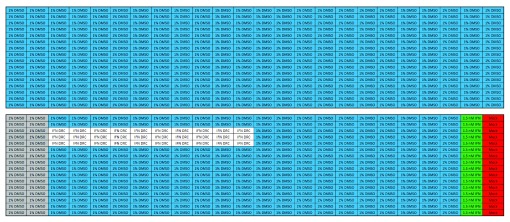
Plate map of the control plates used during the campaign. Upper panel represents the 1% DMSO plate, control of the variability among the wells of the plate. Lower panel represents the dose-response of the reference compound Interferon α 2A, which serves as control of the entire run, where: 1% DMSO vehicle treated ZIKV infected cells (blue), representing the samples; 1.5 nM IFNα2A treated ZIKV infected cells (positive control) (green); Mock infected cells (red); 1% DMSO treated ZIKV infected cells (negative control) (grey).

NA values of the reference compound dose-response curve were processed with the Graphpad Prism software, version 6, for generation of sigmoidal dose-response (variable slope) non-linear curve fitting and determination of the EC _50_ values, defined as the effective concentration resulting in a 50% inhibition of ZIKV infection. The statistical validity of the Zika virus high content screening was determined by calculating the Z'-factor ^27^ using the infected Huh7 treated with 1% DMSO or IFNα2A as negative and positive controls, respectively. As quality control of the screenings, a 1% DMSO plate and two IFNα2A dose-response curve plates were performed in each run ( Figure 2).

### Hit selection criteria

Were considered as hits, compounds that presented both normalized activity (see formula above) and cell ratio (number of cells of the tested compound divided by the mean of 1% DMSO-treated cells) equal or superior of 50%.

### Activity confirmation in dose-response curves

To confirm the compound activity against Zika viruses, the selected hits from both primary screenings were tested in a 9 point DRC, with 2-fold serial dilutions starting at 50 µM, using the same assay and data analysis described for the primary screening. The EC _50_ value was used to evaluate compound activity. The CC _50_ value, defined as the compound concentration resulting in a 50% reduction in cell viability compared with the infected IFNα2A treated cells, was used to evaluate cell toxicity. The compounds that presented the Selectivity Index (SI), which is calculated as SI = CC _50_/EC _50_, equal or higher than 1 and that reached at least 50% of maximum activity were considered as confirmed hits. Here we describe a high content screening methodology for the discovery of inhibitors of ZIKV infection applied in a drug repurposing context. This assay was used to screen a library of FDA-approved drugs, resulting in the identification of five compounds with selective activity against ZIKV in human cells.

## Results

### Assay development

The first step to develop the high content Zika virus screening assay was to adapt the ZIKV to infect a suitable cell line, in this case the human cell line Huh7, in 384-well plates. The optimal cell density, virus MOI and necessary period of time for the efficient viral infection in the host cell were determined. For this propose the Huh7 cells were seeded in four different densities (2×10 ^4^, 4×10 ^4^, 6×10 ^4^ and 8×10 ^4^ cells/mL), combined with three different MOI (0.25, 0.5 and 1) for 2, 3 or 4 days, using mock-infected Huh7 cells as controls. At the assay endpoint, all conditions were submitted to indirect immunofluorescence using as primary antibody the monoclonal mAb 4G2, which recognizes the E protein of flaviviruses, to detect the infected cells. Images were randomly acquired from all conditions and submitted to High Content Analysis for the determination of the infected and non-infected cells populations, followed by the determination of the infection ratio (ratio of infected cells to the total number of cells) and the cytotoxicity. Figure 1 shows a representation of the methodology employed to detect the viral infection in host cells. After analyzing the data, the cell density of 6×10 ^4^ cells/mL, MOI = 0.5 and 72 h of infection were selected as the best conditions for virus infection ( Table S1), presenting the highest infection ratio, varying from 60–90% and cell ratio combined with lowest variation of infection in 384 plates, with a coefficient of variation below 10%.

### Human α Interferon 2A as reference compound and assay validation

**Figure 3.**
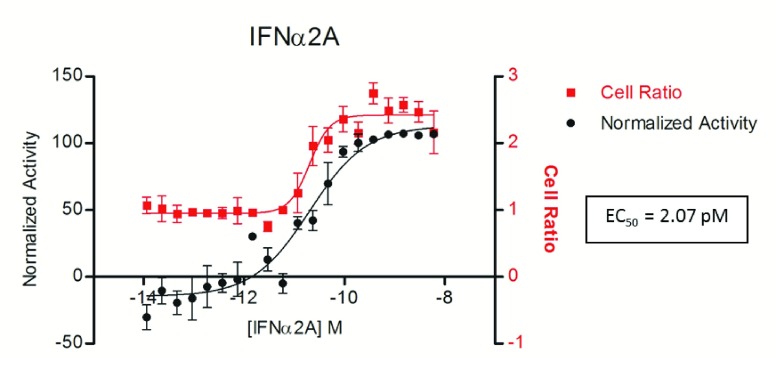
Dose-dependent activity of Interferon α 2A (IFNα2A) against Zika infection. ZIKV infected cells were treated with different doses of IFNα2A in a dose-response curve. After 72 h of incubation the cells were submitted to indirect immunofluorescence assay and the infection ratio determined. The data was normalized with the controls and the resultant normalized activity used to plot a sigmoidal dose-response curve (variable slope). The effective concentration resulting in a 50% inhibition of ZIKV infection (EC _50_) of 2.07 pM was obtained and the concentration of 1.5 nM defined as the effective concentration (capable of eradicating the infection). The cell ratio (number of cells of the tested compound divided by the mean of 1% DMSO-treated cells) is represented in red and the normalized activity in black.

The Interferon α 2A was previously reported to have anti-flaviviral activity ^16, 17^ and, for this reason it was chosen as the reference compound in this assay. The antiviral activity of IFNα2A in ZIKV-infected Huh7 cells was verified in dose-response curves and. After 72 h, the infection level was determined by indirect immunofluorescence (IF) and the extracted data were analyzed and used to plot a sigmoidal dose-response curve ( Figure 3). The EC _50_ of 2.07 pM and the minimal effective concentration (capable of eradicating the infection) of 1.5nM were determined for IFNα2A against ZIKV. As can be observed in Figure 3, IFNα2A activity can also protect against the ZIKV cytopathic effect, which leads to cell lysis; thus, the ratio between the number of cells in treated wells and the number of cells in non-treated wells (defined as the cell ratio) increases in dose-dependent manner to the concentration of IFNα2A, indicating the interferon capacity of protecting the host cells from lysis due to ZIKV infection.

**Figure 4.**
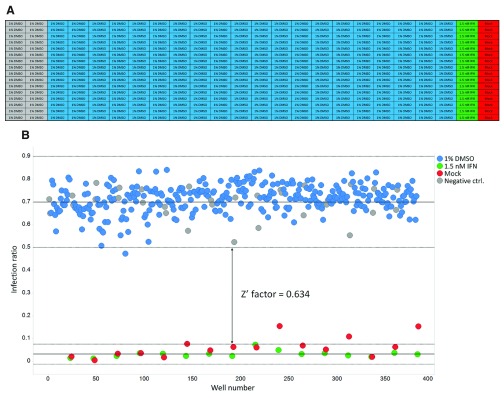
Scatter plot and calculated Z'-factor of the image-based Zika virus high-throughput assay. **A**) Layout of the validation plate. **B**) Scatter plot of the infection ratio (number of infected cells divided by the total number of cells) among the validation plate. Dots represent each single tested well and colors represent different treatments, where: 1% DMSO vehicle treated ZIKV infected cells (blue), representing the samples; 1.5 nM IFNα2A treated ZIKV infected cells (positive control) (green); Mock infected cells (red); 1% DMSO treated ZIKV infected cells (negative control) (grey).

The final step of the assay validation was the evaluation of the Z’-factor ^18^ for ZIKV infection in Huh7 using IFNα2A as the reference compound. Figure 4A shows a representation of the assay performed, where cells viruses and the reference compound where dispensed following the designed 384-well plate template. The assay resultant data were used to generate a scatter plot and a Z'-factor ^18^ of 0.63 was obtained ( Figure 4B).

### Screening of the NIH clinical collection compounds library

**Figure 5.**
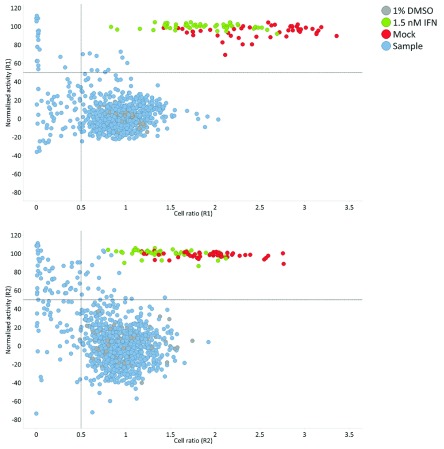
Activity profile of the NIH Clinical Collection library anti-Zika virus high content primary screening. In two independent runs, the compounds selected as hits (normalized activity ≥ 50% and cell ratio ≥ 0.5) are located in the right superior quadrant. Dots represent each single tested well and colors represent different treatments, where: ZIKV infected cells treated with different compound samples (blue); 1.5 nM IFNα2A treated ZIKV infected cells (positive control) (green); Vehicle DMSO 1% treated mock infected cells (red); 1% DMSO treated ZIKV infected cells (negative control) (grey). The table in the right summarizes the results obtained both runs.

**Figure S1.**
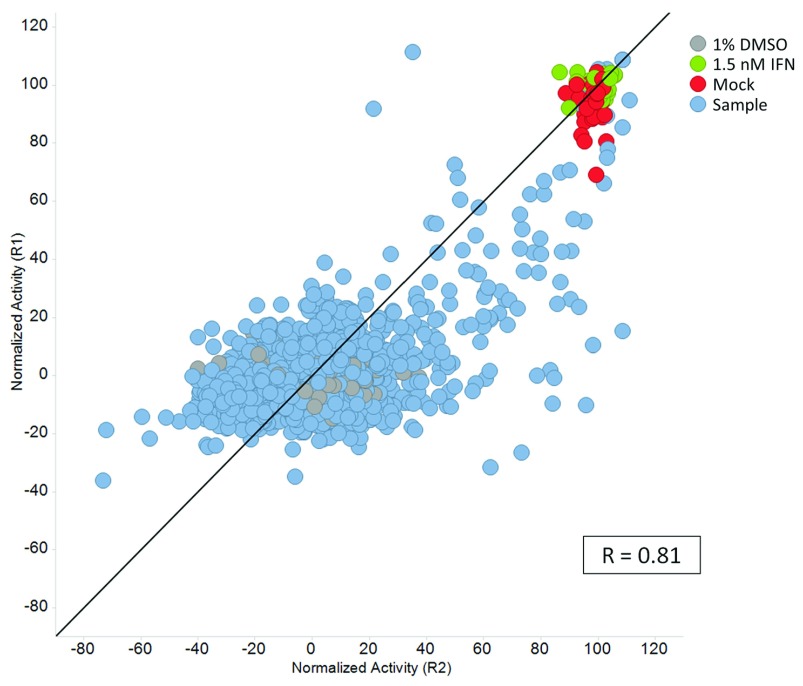
Two independent screenings of the NIH Clinical Collection in the Zika high content assay show a high degree of correlation. Normalized activity of the samples and controls were plotted in Spotfire software (Tibco) and the correlation coefficient (R) of the two runs calculated using Pearson test in GraphPad Prism software. Dots represent each single tested well and colors represent different treatments, where: ZIKV infected cells treated with different compound samples (blue); 1.5 nM IFNα2A treated ZIKV infected cells (positive control) (green); Vehicle 1% DMSO treated mock infected cells (red); 1% DMSO treated ZIKV infected cells (negative control) (grey).

The screened library consisted of725 compounds from a collection of chemically diverse FDA-approved drugs with known and unknown mechanisms of action. The entire library was screened at 20 µM against ZIKV infecting Huh7 cells, using IFNα2A at 1.5 nM as the reference drug and 1% DMSO (vehicle)-treated infected cells as negative controls. As quality control of the assay, two dose-response curves of the reference compound and a 1% DMSO plate were performed ( Figure 2). The library was screened in two independent experiments, and the correlation coefficient (R) of 0.81 obtained, which was determined for the normalized activity of each compound between the first (R1) and the second (R2) screens, including compounds and controls ( Figure S1). The mean Z'-factor of the screenings were 0.74±0.06 for R1, and 0.56±0.09 for R2 ( Figure 5). Out of 725 triaged compounds, 12 and 25 compounds were selected as hits for run 1 and run 2 respectively ( Figure 5), resulting in a hit rate of 4%. As the total number of hits was low, all the hits from both primary screenings were selected for confirmation and further testing by dose-response curves.

### Discovery of new drugs with anti-Zika activity

The compounds selected as hits in the primary screenings were tested in dose-response to confirm their activity and obtain data regarding the maximum activity, selectivity and EC _50_. From 29 selected samples in the primary screening, 22 (76%) presented an SI ≥ 1 and maximum activity ≥ 50%, and were considered confirmed ( Table 2 and Table S2).

From the 22 confirmed compounds, five were selected for further analysis, based on the SI, maximum activity and EC _50_ ( Table 2). These five hits are Lovastatin (Pubchem CID: 53232), an hypolipidemic agent; 5-Fluorouracil (Pubchem CID: 3385), a drug used in the treatment of cancer and that acts through irreversible inhibition of thymidylate synthase; 6-Azauridine (Pubchem CID: 5901), an antineoplastic and antipsoriatic agent, a broad-spectrum antimetabolite that is known to inhibit both DNA and RNA virus multiplication (orotic acid pathway); Palonosetron (identified as hydrochloride salt) (Pubchem CID: 6337614), a 5-HT _3_ antagonist with antiemetic properties, used in the treatment of chemotherapy-induced nausea and vomiting; and Kitasamycin (Pubchem CID: 44634697), a macrolide antibiotic (Josamycin family) with antimicrobial activity against a wide spectrum of pathogens.

There are numerous studies showing antiviral or activity of anticancer drugs ^19^ and of macrolides ^20^. Few papers suggest that statins exhibit anti-inflammatory and antiviral effects *in vitro* ^21^. Recently, Palonosetron anti-ZIKV activity was also verified in an assay similar to the described in this work ^22^.

Raw data of 'identification of drugs and promising starting points for drug discovery from an FDA-approved library' The raw data supporting the findings described in the paper are provided. Click here for additional data file. Copyright: © 2016 Pascoalino BS et al. 2016 Data associated with the article are available under the terms of the Creative Commons Zero "No rights reserved" data waiver (CC0 1.0 Public domain dedication).

## Discussion

The advent of Zika virus infections and its fast spreading across the globe, together with reported association of the ZIKV with severe birth defects, including microcephaly and Guillain-Barré syndrome, has raised attention for the importance of searching for mechanisms of control the disease that go beyond vector surveillance and palliative supporting treatment to ease symptoms. Although the exact causes of microcephaly are still unknown, new data suggest that it may be caused by intrauterine infection during the development of the brain ^23– 26^. Additionally, other studies have shown in animal models that ZIKV is able to infect the placenta and cross it to infect the fetal brain ^5, 27, 28^.

Recent data from World Health Organization reported that in the last years 61 countries and territories presented mosquito-borne transmission of Zika. From these, 13 countries or territories described cases of microcephaly and other central nervous system malformations potentially associated with Zika virus infection. In addition, studies from 10 different countries have reported evidence of person-to-person transmission of Zika virus, probably via a sexual route ^29^, indicating that Zika may not be restricted only to the tropical and sub-tropical areas where the mosquitoes of the genus *Aedes* sp. is found.

New studies demonstrated that plasma immune to Dengue viruses showed substantial cross-reaction to ZIKV, including being capable to initiate ADE of ZIKV infection ^7^, which could, at least partially, explain the huge increment in the number of reported Zika virus infections after Dengue outbreaks and in areas where Dengue virus is prevalent. Moreover, this cross-reactivity of the anti-Dengue sera with Zika viruses could be a risk point for the newly developed anti-Dengue vaccines ^8, 9^.

In the present work, we developed a fast, robust and reliable technology of high content screening assay for Zika virus. This novel methodology identified five promising compounds ( Table 2), among 725 FDA-approved compounds from the NIH Clinical Collection compound library. Two of these compounds were previously described as having anti-ZIKV activity. The 6-Azauridine, which has been reported to have anti-flaviviral activity against 11 members of the flaviviral family, including Zika virus ^17^, and Palonosetron in a similar assay described in this work ^22^. In fact, the detection of hit compounds with previously described anti-ZIKV activity in the screened library validates this approach and demonstrates that the assay is useful for the discovery of novel compounds capable of inhibiting ZIKV infection. It also reinforces that these compounds have promising activity against ZIKV and were able to stand scrutiny of two different screening assays. Conversely, compounds that were recently reported with anti-ZIKV activity ^30^ such as Azathioprine, Dactinomycin, Digoxin, Mebendazole and Mefloquine, presented toxicity higher than 50% in our assay and Clofazimine, Mercaptopurine, Methoxsalen and Sertraline-HCl, which presented activity lower than 50% in our assay. This suggests that these compounds might have a narrow spectrum of activity against some but not all ZIKV isolates. Furthermore, the assay here described is also capable of identifying slowly acting drugs, which demand extended exposure to manifest their effect.

The clear advantage of this screening is the fact that the assay covers the viral entry, RNA synthesis and viral egress of the host cell, since the Huh7 are exposed to Zika virus for 72 h, respecting the viral biology during the infection of the host.

All the five herein identified active compounds are currently marketed drugs for distinct treatments. The molecular structure and pharmacokinetics data of the compounds are summarized in Table 1 and Table 2. Lovastatin belongs to the family of statins, which are widely used for lowering cholesterol in patients with hypercholesterolemia, to reduce risk of cardiovascular disease. A clinical trial tested the efficacy of the treatment of Dengue-infected patients with Lovastatin ^31^, since the endothelial stabilizing effects of statins could decrease Dengue-related vasculopathy. Although Lovastatin anti-flaviviral activity was already reported in hepatitis C virus ^32^ and Dengue virus ^33, 34^, no evidence of a beneficial effect on any of the clinical manifestations or on Dengue viremia was found. In addition, Lovastatin was reported to attenuate nervous injury in animal model of Guillain-Barré syndrome ^35^.

**Table 1.** Molecular structure and dose-response curve of the five most promising compounds identified in the anti-Zika virus high content screening of the NIH Clinical Collection library.

Chemical Name	Structure	Dose-response curve
Lovastatin	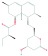	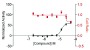
5-Fluorouracil	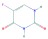	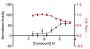
6-Azauridine	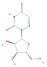	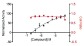
Palonosetron	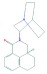	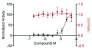
Kitasamycin	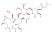	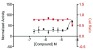

**Table 2.** Summarized chemical and physical properties of the most promising compounds identified with anti-ZIKV activity.

Drug	Palonosetron	6-Azauridine	5-Fluorouracil	Lovastatin	Kitasamycin
Pubchem CID	6337614	5901	3385	53232	44634697
NIH ID	SAM001246791	SAM001246876	SAM002264615	SAM002589963	SAM001246731
EC _50_ (µM)	16.3 ± 7.7	2.3 ± 0.1	14.3 ± 8.6	20.7 ± 8.6	41.7 ± 10.1
CC _50_ (µM)	ND	ND	ND	ND	ND
Max. Activity (% of infection inhibition)	97.7	88.3	57	60.7	69.1
S.I. (CC _50_/EC _50_)	> 3.06	> 33.33	> 2.5	> 2.5	> 1.2
Water Solubility (mg/mL)	Very high	Very high	12.5	0.0004	0.05
M.W. (g/mol)	296.41	245.19	130.08	404.54	828.00
LogP	2.8	-2.1	-0.9	4.3	2.9
HBD	0	4	2	1	3
HBA	2	7	3	5	16
Rot	1	2	0	7	14
TPSA	23.6	132	58.2	72.8	206

EC _50_ (µM)	Max. Activity (% of infection inhibition)	S.I. (CC _50_/EC _50_)	Water Solubility (mg/mL)
<10	>90	>10	High
[10–30]	[75–90]	[2–10]	Medium
>30	<75	<2	Low

5-Fluorouracil is a product of the metabolism of floxuridine, a drug long used in the treatment of diverse types of cancer ^36^. It belongs to a drug class known as antimetabolites, and is a pyrimidine analog that irreversibly inhibits thymidylate synthase, impairing the DNA synthesis. The anti-flaviviral activity of Floxuridine against Dengue and West Nile virus was already reported ^37, 38^, and here we demonstrate that it also has activity against Zika virus.

6-Azauridine is generally administrated as the triacetylated prodrug, Azaribine. 6-Azauridine, is an antimetabolite capable to inhibit both DNA and RNA virus multiplication. 6-Azauridine was withdrawn from clinical use because of the occurrence of arterial and venous thromboembolic episodes in some psoriatic patients ^39^. Early work demonstrated that viruses sensible to 6-Azauridine induced increased levels of uridine kinase, which converts uridine to uridine monophosphate, a nucleotide used in RNA synthesis, that could explain the activity of the 6-Azauridine on such viruses ^40^. More recently, 6-Azauridine was reported to have a broad activity against 11 flaviviruses, including Zika ^17^.

Kitasamycin is a natural product from *Streptomyces narbonensis* that belongs to the macrolide antibiotic class. The compound is a broad spectrum antimicrobial drug against several pathogens, such as Gram positive bacteria, mycoplasma and leptospira. This macrolide binds to bacterial ribosomal RNA and inhibits protein biosynthesis ^41^. Although Kitasamycin is clinically used, this is the first time, to our knowledge, that it has been reported to have antiviral activity.

Palonosetron is a 5-HT _3_ serotonin receptor antagonist used for preventing nausea and vomiting induced by chemotherapeutic agents. Palonosetron anti-ZIKV activity was also reported in a recent work ^22^.

Taking a closer look at the selected compounds, they clearly do not belong to the same class of molecules since their structures are quite different ( Table 1). Moreover, the calculated properties of the molecules also vary widely ( Table 2). Regarding the molecule size, 5-Fluorouracil and 6-Azauridine can be considered small, Palonosetron and Lovastatin are medium-sized while Kitasamycin is a big compound, in terms of drugs. 5-Fluorouracil and 6-Azauridine are hydrophilic while Palonosetron, Lovastatin and Kitasamycin are more lipophilic. 5-Fluorouracil, Lovastatin and Palonosetron have few H bond acceptors and donors, while 6-Azauridine and Kitasamycin have several. Finally, 6-Azauridine and Kitasamycin have a high topological polar surface area (TPSA) while Lovastatin, 5-Fluorouracil and Palonosetron have a low topological polar surface area, compatible with potential brain penetration, which could be a very important feature since the viral infection causes severe damage in the nervous systems under development. These calculated properties allow anticipating very different physico-chemical properties of these hits, likely resulting in very different absorption, distribution, metabolization and excretion profiles for these molecules. These profiles could be considered as advantages or drawbacks in a potential antiviral treatment depending on the target product profile of a Zika treatment and should be used to prioritize these chemotypes for further screening campaigns.

These compounds showed specific activity against a ZIKV isolate originated in Pernambuco-Brazil, one of the states with the highest number of microcephaly and other newborn nervous system malformations reported cases ^42^.

The drugs here described can serve as important starting points for the development of analogs or new molecules for the treatment of Zika. Searching for structural analogs of the five molecules, 4,449 similar structures were identified in Pubchem ( Table S3). Screening these analogs could help gaining knowledge on the structure-activity relationship (SAR), an important step on medicinal chemistry optimization of a lead compound. Moreover, 10 of these analogs are already marketed drugs ( Table S3). We can also consider their historical therapeutic class or mechanism of action as clues to select known chemical entities with similar mechanisms of action to screen against ZIKV. For example, statins or macrolides, widely represented in the pharmacopeia, could be screened in order to identify more potent anti-ZIKV hits. The hits can also be used in target deconvolution studies to identify host molecules involved in ZIKV infection as was already described for other viruses like Chikungunya virus ^43^.

Combining the information generated in this study and the pharmaceutical properties available for the best compounds here identified, we considered Palonosetron as the most promising compound. This drug can be dosed either by oral or intravenous route, in humans its bioavailability is very high (97%) and half-life is very long (40h), making it a good candidate for *in vivo* confirmation. However, its metabolism, albeit low, mainly involves cytochrome P450 2D6. As there is a high interindividual variability in the efficiency and amount of CYP2D6 enzyme produced, it can be anticipated that this drug may be subjected to substantial variation when metabolized in humans. This problem could be addressed in a medicinal chemistry lead optimization project, provided that SAR is observed. However, this drawback did not prevent Palonosetron (commercialized by Esai as Aloxi ^®^) to reach the market. Interestingly, other 5-HT3 antagonists like Dolasetron, Ondansetron, Granisetron, Tropisetron and Alosetron, discovered by different pharmaceutical companies, with similar or different chemotypes, also reached the market. These compounds, generally developed for treatment of chemotherapy-induced nausea have been widely prescribed (off-label) for morning sickness during pregnancy. The possibility to treat pregnant women with this class of compounds is another advantage in Zika infection, albeit their safety profile for newborns is currently controversial ^44, 45^.

In summary, the study developed here describes a high content screening assay which successfully identified five active compounds against Zika virus isolated in an area of high number of reported cases of newborn neural complications. Further investigation is needed to understand the mechanism of action responsible for the inhibition of the Zika virus infection. However, the molecules identified in this study are important starting points, since they can be further optimized to increase the efficiency inhibiting ZIKV infection. Moreover, based on the structure comparison, more than 4000 molecules where identified in the PubChem databank as analogs and structural variants which could be also be tested, and still more specific and potent compounds can still be identified or even designed.

## Data availability

The data referenced by this article are under copyright with the following copyright statement: Copyright: © 2016 Pascoalino BS et al.

Data associated with the article are available under the terms of the Creative Commons Zero "No rights reserved" data waiver (CC0 1.0 Public domain dedication). 

F1000Research: Dataset 1. Raw data of 'identification of drugs and promising starting points for drug discovery from an FDA-approved library', 10.5256/f1000research.9648.d137642 ^46^

## Figures and Tables

**Table S1.** Adaptation of the ZIKV infection in Huh7 hepatoma cells regarding the cell density, multiplicity of infection and infection time.

MOI 0.25												
MOI 0.5												
MOI 1												
Mock infected												
	48 h				72 h				96 h			
	Cell ratio	Cell ratio SD	Infection Ratio	Infection Ratio SD	Cell ratio	Cell ratio SD	Infection Ratio	Infection Ratio SD	Cell ratio	Cell ratio SD	Infection Ratio	Infection Ratio SD
2×10 ^4^ cell/mL	0.675	0.074	0.044	0.018	0.673	0.060	0.206	0.027	0.502	0.057	0.606	0.100
2×10 ^4^ cell/mL	0.862	0.096	0.069	0.029	0.745	0.047	0.240	0.032	0.430	0.069	0.550	0.094
2×10 ^4^ cell/mL	0.587	0.106	0.188	0.092	0.767	0.039	0.259	0.109	0.158	0.048	0.488	0.181
4×10 ^4^ cell/mL	0.873	0.127	0.097	0.046	0.739	0.031	0.271	0.127	0.508	0.079	0.628	0.053
4×10 ^4^ cell/mL	0.909	0.104	0.084	0.027	0.756	0.045	0.392	0.216	0.533	0.066	0.544	0.120
4×10 ^4^ cell/mL	0.997	0.094	0.113	0.039	0.751	0.035	0.550	0.217	0.248	0.086	0.575	0.097
6×10 ^4^ cell/mL	0.974	0.176	0.085	0.022	0.905	0.081	0.780	0.130	0.498	0.060	0.567	0.063
6×10 ^4^ cell/mL	1.059	0.172	0.096	0.029	0.903	0.055	0.836	0.075	0.526	0.035	0.564	0.043
6×10 ^4^ cell/mL	0.839	0.243	0.131	0.171	0.863	0.066	0.800	0.132	0.446	0.061	0.578	0.068
8×10 ^4^ cell/mL	1.038	0.148	0.141	0.039	0.912	0.045	0.548	0.256	0.536	0.048	0.536	0.061
8×10 ^4^ cell/mL	1.065	0.136	0.092	0.031	0.896	0.057	0.537	0.233	0.521	0.038	0.554	0.048
8×10 ^4^ cell/mL	0.926	0.111	0.074	0.038	0.884	0.064	0.430	0.249	0.505	0.029	0.560	0.045

**Table S2.** Summarized information of the identified compounds in the primary screening of the NIH Clinical Collection library not selected for further analysis.

NIH ID#	EC _50_ (µM)	CC _50_ (µM)	Max. Activity (%)	SI
SAM002589981	22 ± 8	38.3 ± 8.7	101.7	1.74
SAM002564206	14.7 ± 4.2	21.3 ± 12.3	98.5	1.87
SAM002564189	19.3 ± 5.5	11.7 ± 11	88.9	0.6
SAM002554886	14.7 ± 3.1	34 ± 11.3	76.1	3.01
SAM002548975	12.3 ± 5.7	43.7 ± 9.3	92.9	3.54
SAM002548938	21.3 ± 10	31.3 ± 15.3	96.6	1.47
SAM002264636	19.7 ± 4.5	28.3 ± 17.6	87.1	1.44
SAM001247107	14 ± 0	19.3 ± 9.7	92.2	1.38
SAM001247103	8 ± 2.6	8 ± 3	101.7	1
SAM001247083	21.5 ± 0.7	16.5	68.5	1.34
SAM001247075	12 ± 1.7	33.7 ± 10.8	95.6	2.8
SAM001247056	14.3 ± 3.5	8.7 ± 1.2	99.8	0.6
SAM001247054	10.3 ± 3.1	9 ± 4.4	101.7	0.87
SAM001247052	9.7 ± 1.5	14.7 ± 2.5	92.6	1.51
SAM001247038	17.3 ± 4.9	31.3 ± 15	88.8	1.8
SAM001246989	15.3 ± 5.8	13.3 ± 7.6	101.7	0.87
SAM001246979	17 ± 4.2	23.5 ± 13.6	68.9	1.38
SAM001246977	17 ± 3.6	24.3 ± 3.1	101.3	1.43
SAM001246883	13 ± 1	32 ± 14	83.6	2.46
SAM001246690	14.5 ± 0.7	26.5 ± 0.7	95.1	1.82
SAM001246686	ND	ND	69.7	ND
SAM001246644	5.3 ± 1.5	29 ± 2	72	5.44
SAM001246622	13.3 ± 5.8	20.3 ± 2.9	90.2	1.75
SAM001246545	10.7 ± 2.1	20.7 ± 4.1	88.7	1.94

**Table S3.** Analogs compounds and Medications of the compounds identified with anti-ZIKV activity.

		Analogs in Pubchem	
INN	Pubchem CID	Similar compounds	Medications
Lovastatin	53232	2361	Mevastatin L669262 6-Hydroxyisocompactin Monacolin J, L, M Simvastatin Wuxistatin
5-Fluorouracil	3385	71	Methimazole
6-Azauridine	5901	127	Decitabine Cytarabine
Palonosetron	6337614	419	none
Kitasamycin	44634697	1471	Tylosin
		4449	
