# Supplementary material for: Zika antiviral chemotherapy: identification of drugs and promising starting points for drug discovery from an FDA-approved library
Source: F1000Res. 2016 Oct 14;5:2523. [Version 1] doi: 10.12688/f1000research.9648.1 (PMC5112578; doi:10.12688/f1000research.9648.1)
Supplement: Peer review history

# Peer review: Referee response for version 1

**DOI:** 10.5256/f1000research.10396.r17530

**Published:** 2016-11-15

**Authors:** Tom von Geldern

**Affiliations:** 1 Embedded Consulting, Illinois, IL, USA

Infection by Zika virus (ZIKV) is an emerging global health crisis, and as of today there are no approved therapeutic interventions are available for patients. In a search for novel anti-ZIKV agents which could be moved quickly into clinical use, the authors have applied their well-established high-throughput, high-content assay platform to develop a ZIKV assay, and have applied this to evaluate a library of FDA-approved drugs. Building a high-quality high-content assay is a complex exercise, but this team is quite experienced, and has had particular success in the past with other tropical infectious agents.

 Starting from a library of 725 compounds, a series of staged triage steps leads to the identification of 5 validated hits. While the established mechanisms for the majority of these hits (anti-cancer, antibiotic) are recognized sources of anti-parasitic leads, two are quite unexpected. Because they have been sourced from a collection of approved drugs, all should be able to move rapidly into in vivo proof-of-concept studies. Additionally, they might serve as starting points for further optimization by drug discovery teams; the sharing of these lead structures is a particularly altruistic decision on the part of the team.

 The work reported here is very similar to that reported recently by another multi-national, multi-disciplinary consortium ^1^, who screened a nearly-identical collection in a similar manner. Interestingly, there was very little overlap in the hit-sets identified by these two teams. The origin of this difference is unclear, though the two groups used ZIKV from different sources; this may suggest that anti-viral activity is strain-dependent, complicating the development of agents with broad-based activity. It would be very interesting to have each team cross-test the other's set of leads in their own assay format.

I have read this submission. I believe that I have an appropriate level of expertise to confirm that it is of an acceptable scientific standard.

## References

[1] BarrowsNJCamposRKPowellSTPrasanthKRSchott-LernerGSoto-AcostaRGalarza-MuñozGMcGrathELUrrabaz-GarzaRGaoJWuPMenonRSaadeGFernandez-SalasIRossiSLVasilakisNRouthABradrickSSGarcia-BlancoMA: A Screen of FDA-Approved Drugs for Inhibitors of Zika Virus Infection. *Cell Host Microbe*.2016;20(2) : 10.1016/j.chom.2016.07.004 259-70 10.1016/j.chom.2016.07.004 27476412PMC4993926

# Peer review: Referee response for version 1

**DOI:** 10.5256/f1000research.10396.r17012

**Published:** 2016-10-26

**Authors:** Paul S Anderson

**Affiliations:** 1 Independent Pharmaceuticals Professional, Lansdale, PA, USA

The authors describe a cell based assay suitable for screening compounds for activity against Zika virus. The assay is compatible with high content screening methodology and was used in this mode to screen a library of FDA-approved drugs. Palonosetron, 6-Azauridine, 5-Fluorouracil, Lovastatin and Kitasamycin were identified as low micromolar hits. The mechanism(s) for inhibition of the Zika virus infection was not identified.

I have read this submission. I believe that I have an appropriate level of expertise to confirm that it is of an acceptable scientific standard.
